# The kidney injury biomarker profile of patients with lupus nephritis remains unchanged with the second-generation calcineurin inhibitor voclosporin

**DOI:** 10.3389/fneph.2025.1540471

**Published:** 2025-03-17

**Authors:** Biff F. Palmer, James A. Tumlin, Jai Radhakrishnan, Linda M. Rehaume, Jennifer L. Cross, Robert B. Huizinga

**Affiliations:** ^1^ Department of Internal Medicine, University of Texas Southwestern Medical Center, Dallas, TX, United States; ^2^ NephroNet Clinical Research Consortium, Atlanta, GA, United States; ^3^ Division of Nephrology, Columbia University Medical Center, New York, NY, United States; ^4^ Aurinia Pharmaceuticals Inc., Edmonton, AB, Canada

**Keywords:** lupus nephritis, voclosporin, biomarker, nephrotoxicity, calcineurin inhibitor

## Abstract

**Objectives:**

Kidney injury in patients with lupus nephritis (LN) results in pro-fibrotic biomarker expression, a manifestation also observed with calcineurin inhibitor (CNI) therapy. The second-generation CNI, voclosporin, is approved in the United States and Europe for the treatment of patients with active LN in combination with background immunosuppression, based on successful outcomes from the global phase 2 AURA-LV and phase 3 AURORA 1 studies, which demonstrated the efficacy of voclosporin across diverse racial and ethnic populations, and encompassing multiple biopsy classes of LN, alongside a favorable safety profile. This *post hoc* analysis examined changes from baseline levels of serum and urinary biomarkers, including pro-fibrotic biomarkers, in a cohort of patients from the parent AURORA 1 study

**Methods:**

Samples were analyzed from a cohort of patients in AURORA 1 treated with voclosporin (23.7 mg twice daily, n=57) or placebo (n=59) in combination with mycophenolate mofetil (MMF) and low-dose glucocorticoids, including in a subgroup of patients that experienced a ≥30% decline from baseline in estimated glomerular filtration rate (voclosporin, n=26; placebo, n=20).

**Results:**

The addition of voclosporin to MMF and low-dose glucocorticoids for the treatment of LN did not result in significant differences in normalized urinary concentrations of KIM-1, TGF-β1, MCP-1, or NGAL, biomarkers indicative of renal fibrosis and kidney damage, when compared to MMF and low-dose glucocorticoids alone.

**Conclusion:**

These findings further support the safety of voclosporin for the treatment of LN in adult patients.

**Clinical Trial Registration:**

ClinicalTrials.gov **, identifier NCT03021499; EudraCT, identifier 2016-004045-81.**

## 1 Introduction

Lupus nephritis (LN) is one of the most severe clinical manifestations of systemic lupus erythematosus (SLE) and is a major cause of morbidity and mortality in this patient population, occurring in around half of the patients diagnosed with SLE. Furthermore, 40% of patients with LN are thought to experience varying degrees of renal impairment, with 10-30% of patients with LN progressing to chronic kidney failure within 15 years of diagnosis (1–4). The past five decades have witnessed significant advances in treatment options for SLE patients, including the introduction of calcineurin inhibitors (CNIs) to the immunosuppressive regimen, a course of therapy associated with improved prognosis (5, 6). Early studies with first-generation CNIs (cyclosporine A [CsA] and tacrolimus [TAC]) report improved efficacy in LN patients (7–9). Recently, the second-generation CNI, voclosporin, has shown to be a promising alternative to first-generation CNIs in the treatment of LN (10, 11).

Voclosporin, in combination with a background immunosuppressive regimen, is the first approved oral therapy for adult patients with active LN in the United States and Europe based on safety and efficacy outcomes from two pivotal, global, clinical studies, the AURA-LV phase 2 and AURORA 1 phase 3 studies (10, 11). Moreover, an additional two years of safety and efficacy data were reported for the phase 3 AURORA 2 continuation study (12). Outcomes demonstrated improved renal response rates in patients treated with voclosporin, compared to the control arm of placebo, both combined with mycophenolate mofetil (MMF) and low-dose glucocorticoids. Voclosporin demonstrated efficacy across diverse racial and ethnic populations, and multiple biopsy classes of LN. Improved kidney function, indicated by significant decreases in proteinuria with stable estimated glomerular filtration rate (eGFR), without a clinically meaningful impact on serum electrolytes, was accompanied by safety profiles, comparable to patients treated with MMF and low-dose glucocorticoids alone, following three years of treatment (10–12).

CNI immunosuppression derives from reduced T-cell activation via calcineurin inhibition. Distinct from immunosuppression, CNI use is associated with nephrotoxicity, resulting in considerable impact on the clinical management of patients requiring CNI therapy, including kidney and non-kidney solid organ transplantation patients. Classed as acute or chronic, dependent on the reversibility of functional changes, nephrotoxicity observed with long-term administration of CsA and TAC is associated with progressive kidney damage following solid organ transplantation (13–16).

Kidney fibrosis, in particular “striped fibrosis”, is a well-described yet unique form of nephrotoxicity associated with CNI use (17). Whilst the mechanism is not fully understood, chronic ischemia along the medullary rays of the kidney may contribute to tubular drop-out and fibrosis. Other factors may include direct stimulation of transforming growth factor beta (TGF-β) by renal tubular epithelium (18). A number of pro-fibrotic and pro-inflammatory markers, including kidney injury molecule-1 (KIM-1), neutrophil gelatinase-associated lipocalin (NGAL), monocyte chemoattractant protein-1 (MCP-1) and TGF-β1 are linked to kidney injury and fibrosis in patients with LN and in patients exposed to CNIs (19–21). Thus, it would be beneficial for a CNI to exhibit efficacy whilst limiting treatment-related adverse events (AEs). Electrolyte derangement is another indicator of kidney damage, with hypomagnesemia and hyperkalemia often evident in patients receiving CNI therapy.

Based on AURORA 1, where improved urine protein creatinine ratio (UPCR) was observed without a clinically meaningful impact on serum electrolytes, including magnesium and potassium, the current study reports on a *post hoc* analysis of biomarker profiles in a sample of AURORA 1 patients with active LN. Pro-fibrotic biomarker expression was evaluated in LN patients treated with voclosporin or placebo, both in combination with MMF and low-dose glucocorticoids. Additionally, to rule out dietary impact on the levels of serum electrolytes, urinary electrolytes were analyzed in both treatment arms.

## 2 Materials and methods

### 2.1 AURORA 1 study design

Details of the AURORA 1 study have been reported previously, however, the inclusion and exclusion criteria for AURORA 1 can also be found in the Supplementary Information (**Supplementary Table S1**) (11).

Briefly, patients with biopsy-proven (Class III, IV or V ± III/IV) active LN according to American College of Rheumatology (ACR) criteria, proteinuria ≥1.5 g/g (≥2 g/g for pure Class V), and eGFR >45 mL/min/1.73 m^2^, were eligible to enroll and were randomized to the voclosporin arm (23.7 mg twice daily [BID], n=179) or control arm receiving placebo (n=178) for the study duration (52 weeks). All patients received MMF (target dose 1 g, BID) and low-dose oral glucocorticoids (tapered over 16 weeks to 2.5 mg/day). Given the known hemodynamic effects of CNIs, eGFR was monitored and guidance provided on study drug dose modification for patients experiencing decreases.

Patients provided written informed consent prior to trial-related activities, in accordance with the Declaration of Helsinki and International Council for Harmonisation of Technical Requirements for Pharmaceuticals for Human Use and Good Clinical Practice guidelines. An Institutional Review Board or Independent Ethics Committee at each site approved the informed consent form and protocol. AURORA 1 is registered with ClinicalTrials.gov (NCT03021499) and EudraCT (2016–004045–81).

### 2.2 Patient selection

For this current study, a random sample of 60 patients per treatment arm was selected with approximately 50% treatment responders, 50% non-responders, and all patients that experienced a ≥30% decrease from baseline in eGFR (confirmed based on two consecutive eGFR measures). Treatment responders were defined as those with >50% reduction from baseline in UPCR at Week 52; all patients not achieving at 50% reducing in UPCR, including patients with missing data, were considered non-responders. Samples obtained at baseline and end of treatment visits were analyzed; the number of patients with samples for each analysis is detailed in **Supplementary Figure S1**.

### 2.3 Biomarker analysis

Biomarker analysis of urine and serum samples was carried out by Myriad RBM (Austin, Texas, USA). Biomarker analysis included several inflammatory cytokines and chemokines (**Supplementary Table S2**). Change from baseline (CFB) was calculated by subtracting a patient’s baseline from their EoT value, providing an absolute value. All urinary biomarkers were normalized to urinary creatinine to account for changes in urine concentration. All analyses are presented here as Least Square (LS) means.

### 2.4 Creatinine and electrolyte analysis

Urinary electrolyte analysis was performed at Medpace, Inc. (Stirling, UK) and comprised 24-hour urine samples analyzed for creatinine, magnesium, potassium and sodium and the change in each from baseline. As well as data for 24-hour urine electrolyte excretion rates, changes in absolute concentration of each urinary electrolyte were also analyzed.

### 2.5 Statistical analysis

Statistical analysis of biomarker and electrolyte expression was performed by comparing voclosporin and control treatment arms, using data from an all-patient cohort (which included all responders, non-responders and patients who experienced a ≥30% decrease in eGFR; urine biomarkers n=50; serum biomarkers n=108; electrolytes n=116), a responder cohort (urine biomarkers n=27; serum biomarkers n=50; electrolytes n=56), a non-responder cohort (urine biomarkers n=23; serum biomarkers n=58; electrolytes n=60) and a cohort of all patients who experienced a ≥30% decrease in eGFR in AURORA 1 (urine biomarkers n=21; serum biomarkers n=42; electrolytes n=46). The study was not powered to detect differences between cohorts.

Each parameter was summarized at baseline using a general linear model incorporating a covariate for treatment arm. LS means and standard error (SE) were calculated from this model. End of treatment and CFB data were analyzed using a similar model that included a covariate for the baseline of the applicable parameter. LS means and SE for results within each treatment arm were calculated. This model assessed differences in the CFB to EoT between arms, summarized using the estimate of treatment difference, a SE, and 95% confidence interval (CI).

## 3 Results

Of the random sample of 120 patients from AURORA 1, four patients were excluded because they did not have urinary or serum samples. In total, 116 patients (voclosporin arm, n=57; control arm, n=59) were included in the study with 50 patients as part of the urinary biomarker analysis, 108 patients contributing to the serum biomarker analysis and 116 patients with samples for the electrolyte analysis.

For patients in the eGFR decrease cohort, median (interquartile range [IQR]) treatment duration was 22.9 (15.79 – 37.43) weeks in the control arm (n=20) and 26.2 (16.71 – 51.14) weeks in the voclosporin arm (n=26). For all other patients, the median (IQR) treatment duration was 52 (50.86, 53.14) weeks in the control arm and 52 (51.14, 53.14) weeks in the voclosporin arm.

### 3.1 Urinary biomarker analysis

Of the 43 urinary analytes assayed, 27 had consistently measurable levels. There were no significant changes from baseline to EoT in markers associated with acute nephrotoxicity or kidney fibrosis (KIM-1, MCP-1, NGAL or latency-associated peptide [LAP] TGF-β) when comparing across treatment arms (**Table 1**; **Supplementary Table S3**). This was consistent in responder and non-responder patients (data not shown) as well as in patients who experienced a ≥30% decrease in their eGFR (**Supplementary Table S3**).

**Table 1:** Normalized urinary kidney injury and pro-fibrotic biomarker levels in all-patient and eGFR decrease cohorts.

Analyte	All-patient cohort			eGFR decrease cohort^a^		
	CFB to EoT^b^, LS means (SE)					
	Voclosporin (n=25)	Control (n=25)	Estimate CFB difference vs control (95% CI)	Voclosporin (n=13)	Control (n=8)	Estimate CFB difference vs control (95% CI)
KIM-1	-0.008 (0.0032)	-0.007 (0.0032)	-0.002 (-0.011, 0.008)	-0.006 (0.0060)	0.004 (0.0077)	-0.010 (-0.031, 0.011)
LAP TGF-β1	-0.0018 (0.00029)	-0.0016 (0.00029)	-0.0002 (-0.0010, 0.0007)	-0.0021 (0.00051)	-0.0009 (0.00065)	-0.0013 (-0.0030, 0.0005)
MCP-1	-15.0 (3.30)	-19.1 (3.30)	4.1 (-5.3, 13.5)	-12.0 (3.98)	-1.5 (5.09)	-10.5 (-24.1, 3.1)
NGAL	-0.0 (0.89)	-1.1 (0.89)	1.0 (-1.5, 3.6)	1.4 (1.84)	-0.1 (2.34)	1.5 (-4.8, 7.7)

Analysis of patients from each treatment arm of AURORA 1, including patients with a ≥30% decline from baseline in eGFR during the study. Data is presented as the CFB to EoT of LS means (SE) of normalized levels of urinary analytes. Also presented are estimates of the difference in CFB (95% CI) between the voclosporin and control arms. ^a^eGFR decrease defined as ≥30% reduction from baseline in eGFR during the AURORA 1 study. This study was not powered or designed to detect differences in this cohort. CFB, change from baseline; CI, confidence interval; eGFR, estimated glomerular filtration rate; EoT, end of treatment; IQR, interquartile range; KIM-1, kidney injury molecule-1; LAP TGF-β1, latency-associated peptide of transforming growth factor beta 1; LS, least squares; MCP-1, monocyte chemotactic protein 1; NGAL, neutrophil gelatinase-associated lipocalin SE, standard error.

Of the 27 measurable urinary biomarkers, a change in the baseline expression of only three biomarkers was found to differ significantly between treatment arms at EoT in the all-patient and eGFR cohorts. Whilst decreased levels of eotaxin-1, interleukin (IL)-1β and IL-1α were observed at EoT compared to baseline in all voclosporin-treated patients, in contrast, increased expression of all three biomarkers was observed in all placebo-treated patients. A similar effect was seen with eotaxin-1 and IL-1β in patients that experienced a ≥30% decline in their eGFR (**Supplementary Table S3**). Voclosporin treatment did not impact the CFB expression of any other biomarker when compared to patients in the control arm (**Supplementary Table S3**). Absolute levels of urinary biomarkers can be found in **Supplementary Table S4**.

### 3.2 Serum biomarker analysis

Of the 30 serum biomarkers assessed, 27 had consistently measurable levels and, as with urinary analysis, KIM-1 expression remained unchanged in the serum of any patient cohort (**Supplementary Table S5**). All-patient cohort analyses identified a significant difference in the CFB expression in macrophage migration inhibitory factor (MIF) in the voclosporin arm, compared to the control arm, with a smaller decrease from baseline observed in voclosporin-treated patients than the decrease seen in the control arm (**Supplementary Table S5**). In patients who experienced a ≥30% decrease in eGFR, a significantly different change in baseline expression was found only for B lymphocyte chemoattractant (BLC), with an increase from baseline in the voclosporin treatment arm compared to a decrease from baseline in the control arm at EoT (**Supplementary Table S5**).

### 3.3 Serum and urinary electrolyte analysis

Previously published AURORA 1 data reports stable electrolytes throughout the study with no observed evidence of tubular toxicity (11). Mean values for electrolytes were stable throughout the study; importantly, magnesium (Baseline [mean ± SD], 2.093 ± 0.2074 mg/dL; EoT [mean ± SD], 2.043 ± 0.2115 mg/dL) and potassium (Baseline [mean ± SD], 4.089 ± 0.4857 mmol/L; EoT [mean ± SD], 4.183 ± 0.4233 mmol/L) remained within normal limits (magnesium, 1.8 – 2.4 mg/dL; potassium, 3.5 – 5.1 mmol/L). Moreover, there were no significant differences in the fractional excretion (%) of magnesium, sodium, or potassium at EoT observed in this *post hoc* analysis (**Supplementary Table S6**).

## 4 Discussion

The current study assessed urine and serum analytes in a sample of placebo- and voclosporin-treated patients with active LN from the AURORA 1 study, including patients who experienced an eGFR decrease ≥30% during the trial. Voclosporin treatment was not associated with the expression of biomarkers indicative of kidney fibrosis or damage (KIM-1, TGF-β, NGAL or MCP-1) and demonstrated favorable electrolyte homeostasis.

Numerous preclinical studies have reported on the expression of pro-fibrotic biomarkers, such as KIM-1, TGF-β, NGAL or MCP-1, in relation to kidney function. For example, MCP-1, which is found in rat renal interstitial fibroblasts and damaged cortical tubules, shows increased expression with interstitial inflammation and fibrosis, alongside another pro-fibrotic marker, TGF-β (22–24). Increased NGAL expression has also been reported in proximal tubule cells in a mouse model of acute kidney injury (AKI) and in a model of drug-induced nephrotoxicity, with the authors suggesting that NGAL may be suitable as a urinary biomarker in ischemic and nephrotoxic renal injury (25).

Clinically, expression of the aforementioned biomarkers may indicate the progression of kidney disease (CKD). Kidney fibrosis is commonly observed in CKD, and TGF-β is a critical pro-fibrotic molecule which up-regulates matrix protein synthesis, inhibits matrix degradation, and alters the epithelial mesenchymal transition (26). KIM-1 is associated with an increased incidence of interstitial fibrosis. Given that KIM-1 is expressed in regenerating proximal tubules, it has been proposed as a useful biomarker for tubular kidney injury (19, 27–31). Further, KIM-1 levels have been found to be significantly higher in patients with SLE and active kidney disease, compared with patients without renal activity, an association also seen with the biomarkers MCP-1 and NGAL (32–34). Additionally, both MCP-1 and NGAL are correlated with renal decline and allograft failure in transplant patients.

Given the considerable overlap in the expression of KIM-1, MCP-1, TGF-b and NGAL in kidney injury, several studies have looked at the concurrent expression of two or more markers as a more accurate indicator of renal function (30, 35). This includes the Renal Activity Index for Lupus (RAIL) which is based on the expression of six urinary biomarkers including NGAL, MCP-1, and KIM-1 (36).

Over and above the effect seen with kidney injury, the use of traditional CNIs such as CsA and TAC is also known to result in pro-fibrotic marker expression. Studies in rats treated with CsA or TAC have demonstrated renal function decline and interstitial fibrosis accompanied by increased expression of TGF-β, NGAL, MCP-1 and KIM-1, attributed to CNI-induced nephrotoxicity (37–43). Future studies in preclinical animal models could provide insight into the mechanistic differentiation of voclosporin from CsA and TAC. There is also evidence of both CsA and TAC increasing TGF-β production in the kidneys of transplant patients, correlated with diffuse interstitial fibrosis and graft atherosclerosis (44–50). Fibrogenic effects in patients undergoing kidney transplantation with increased serum levels of TAC and proximal tubule injury are also accompanied by increased levels of KIM-1 (19).

The lack of significant changes between treatment arms in urinary or serum levels of any of the biomarkers shown to be associated with kidney injury or fibrosis suggests no clinically meaningful bearing on the biomarkers analyzed, regardless of treatment outcome. Furthermore, the lack of biomarker expression seen in patients treated with voclosporin, MMF and low-dose glucocorticoids also suggests a concurrent lack of acute tubular injury in these patients.

Across the entire patient cohort in this *post hoc* analysis, the change in expression from baseline to EoT of a very small proportion of biomarkers were found to be significantly altered in the urine (eotaxin-1, IL-1α and IL-1β) and serum (MIF and BCL) of voclosporin-treated patients, however, the clinical relevance of these changes is, as yet, undetermined. Previous studies have reported increased eotaxin levels in the plasma of LN patients and SLE patients with organ damage and IL-1β and IL-1 levels are increased in the kidneys of mice with LN (51). Although IL-1α in LN has received limited attention it has been reported to contribute to glomerulonephritis in mice (52). Macrophage migration inhibitory factor has been shown to play a role in kidney dysfunction with possible therapeutic potential in LN patients (53, 54). B lymphocyte chemoattractant may also play a role in SLE and LN pathogenesis, with higher serum levels in patients with SLE, further increased in patients with LN (55). Given that calcineurin is activated by increased intracellular Ca^2+^ concentrations and plays an essential role in many signalling pathways, inhibition by voclosporin will affect many pathways and is likely responsible for the changes observed in other biomarkers.

The stable mean levels of blood pressure and electrolytes observed throughout AURORA 1 is suggestive of a lack of tubular toxicity. These findings were also consistent with results from the phase 2 AURA-LV study, the phase 3 AURORA 2 study, and further confirmed by an integrated data analysis from combined phase 2 AURA-LV and phase 3 AURORA 1 clinical studies (10–12, 56). In contrast, use of traditional CNIs is known to lead to electrolyte and metabolic abnormalities. Indeed, CsA- and TAC-induced immunosuppression has been shown to impact electrolyte levels in kidney transplant patients (57, 58). However, it is possible that dietary supplementation of electrolytes may correct serum electrolyte imbalances. The *post hoc* analysis presented here demonstrates, once again, that voclosporin treatment does not significantly impact serum or urine magnesium, potassium, or sodium levels confirming no impact of dietary supplementation on the previous findings in serum concentrations of electrolytes for this patient cohort. Voclosporin treatment reduced urinary magnesium 24-hour excretion in patients who experienced an eGFR decrease ≥30%, however, as previously mentioned, LS mean levels remained within normal limits. Hypomagnesemia, via renal magnesium wasting, is common in CsA- and TAC-treated patients, presumably due to effects on reabsorption. Hypomagnesemia is also implicated as a significant contributor to nephrotoxicity associated with CsA and TAC and, notably, features on the labeling for both CNIs (15, 58–60). Previously reported study data from AURORA 1 confirmed no unexpected safety signals, unanticipated AEs, or evidence of chronic nephrotoxicity (11).

The analysis presented here includes a small subgroup of AURORA 1 patients treated for up to 52 weeks and results may not be generalizable to the overall population. No trends indicating nephrotoxicity emerged, however, nephrotoxicity associated with traditional CNIs is observed following long-term administration. The 52-week study duration of AURORA 1 limits the ability of these findings to be extrapolated to chronic or long-term exposure effects. Notably, the AURORA 2 clinical trial provides follow up data on up to 3 years of treatment exposure with voclosporin in a larger cohort, demonstrating sustained efficacy and safety, whilst maintaining electrolyte homeostasis (12). Histopathological evaluation after 18 months of treatment in a small subset of patients from AURORA 2 confirmed exposure to study treatment was not associated with nephrotoxicity (12).

In conclusion, voclosporin treatment in LN patients does not induce common indicators of nephrotoxicity such as pro-fibrotic markers or electrolyte imbalance. These data support a much-improved biomarker profile for voclosporin over traditional CNIs.

## Data Availability

The datasets presented in this article are not readily available due to patient privacy reasons. Requests to access the datasets should be directed to LRehaume@auriniapharma.com.

## References

[1] Davidson A. What is damaging the kidney in lupus nephritis? Nat Rev Rheumatol. (2016) 12:143–53. doi: 10.1038/nrrheum.2015.159 PMC4820834 26581344

[2] Fanouriakis A Kostopoulou M Cheema K Anders HJ Aringer M Bajema I. 2019 Update of the Joint European League Against Rheumatism and European Renal Association-European Dialysis and Transplant Association (EULAR/ERA-EDTA) recommendations for the management of lupus nephritis. Ann Rheum Dis. (2020) 79:713–23. doi: 10.1136/annrheumdis-2020-216924 32220834

[3] Rovin BH Adler SG Barratt J Bridoux F Burdge KA Chan TM. KDIGO 2021 clinical practice guideline for the management of glomerular diseases. Kidney Int. (2021) 100:S1–S276. doi: 10.1016/j.kint.2021.05.021 34556256

[4] Parikh SV Almaani S Brodsky S Rovin BH. Update on lupus nephritis: core curriculum 2020. Am J Kidney Dis. (2020) 76:265–81. doi: 10.1053/j.ajkd.2019.10.017 32220510

[5] Tektonidou MG Dasgupta A Ward MM. Risk of end-stage renal disease in patients with lupus nephritis, 1971–2015: A systematic review and bayesian meta-analysis. Arthritis Rheumatol. (2016) 68:1432–41. doi: 10.1002/art.39594 PMC5071782 26815601

[6] Mok CC. Calcineurin inhibitors in systemic lupus erythematosus. Best Pract Res Clin Rheumatol. (2017) 31:429–38. doi: 10.1016/j.berh.2017.09.010 29224682

[7] Szeto CC Kwan BC Lai FM Tam LS Li EK Chow KM. Tacrolimus for the treatment of systemic lupus erythematosus with pure class V nephritis. Rheumatol (Oxford). (2008) 47:1678–81. doi: 10.1093/rheumatology/ken335 18753192

[8] Zavada J Pesickova S Rysava R Olejarova M Horák P Hrncír Z. Cyclosporine A or intravenous cyclophosphamide for lupus nephritis: the Cyclofa-Lune study. Lupus. (2010) 19:1281–9. doi: 10.1177/0961203310371155 20605876

[9] Miyasaka N Kawai S Hashimoto H. Efficacy and safety of tacrolimus for lupus nephritis: a placebo-controlled double-blind multicenter study. Mod Rheumatol. (2009) 19:606–15. doi: 10.3109/s10165-009-0218-5 19688181

[10] Rovin BH Solomons N Pendergraft WF 3rd Dooley MA Tumlin J Romero-Diaz J. A randomized, controlled double-blind study comparing the efficacy and safety of dose-ranging voclosporin with placebo in achieving remission in patients with active lupus nephritis. Kidney Int. (2019) 95:219–31. doi: 10.1016/j.kint.2018.08.025 30420324

[11] Rovin BH Teng YKO Ginzler EM Arriens C Caster DJ Romero-Diaz J. Efficacy and safety of voclosporin versus placebo for lupus nephritis (AURORA 1): a double-blind, randomised, multicentre, placebo-controlled, phase 3 trial. Lancet. (2021) 397:2070–80. doi: 10.1016/S0140-6736(21)00578-X 33971155

[12] Saxena A Ginzler EM Gibson K Satirapoj B Santillán AEZ Levchenko O. Safety and efficacy of long-term voclosporin treatment for lupus nephritis in the phase 3 AURORA 2 clinical trial. Arthritis Rheumatol. (2024) 76:59–67. doi: 10.1002/art.42657 37466424

[13] Naesens M Kuypers DR Sarwal M. Calcineurin inhibitor nephrotoxicity. Clin J Am Soc Nephrol. (2009) 4:481–508. 10.2215/CJN.04800908 19218475 10.2215/CJN.04800908

[14] Nankivell BJ Borrows RJ Fung CL O’Connell PJ Allen RD Chapman JR. The natural history of chronic allograft nephropathy. N Engl J Med. (2003) 349:2326–33. doi: 10.1056/NEJMoa020009 14668458

[15] Farouk SS Rein JL. The many faces of calcineurin inhibitor toxicity-what the FK? Adv Chronic Kidney Dis. (2020) 27:56–66. doi: 10.1053/j.ackd.2019.08.006 32147003 PMC7080294

[16] Hošková L Málek I Kopkan L Kautzner J. Pathophysiological mechanisms of calcineurin inhibitor-induced nephrotoxicity and arterial hypertension. Physiol Res. (2017) 66:167–80. doi: 10.33549/physiolres 27982677

[17] Nankivell BJ P’Ng CH O’Connell PJ Chapman JR. Calcineurin inhibitor nephrotoxicity through the lens of longitudinal histology: comparison of cyclosporine and tacrolimus eras. Transplantation. (2016) 100:1723–31. doi: 10.1097/TP.0000000000001243 27306529

[18] Akool el S Doller A Babelova A Tsalastra W Moreth K Schaefer L. Molecular mechanisms of TGF beta receptor-triggered signaling cascades rapidly induced by the calcineurin inhibitors cyclosporin A and FK506. J Immunol. (2008) 181:2831–45. doi: 10.4049/jimmunol.181.4.2831 18684975

[19] Cosner D Zeng X Zhang PL. Proximal tubular injury in medullary rays is an early sign of acute tacrolimus nephrotoxicity. J Transplant. (2015) 2015:142521. doi: 10.1155/2015/142521 26199736 PMC4495174

[20] Susianti H Wijaya JW Rastini A Handono K Gunawan A Kalim H. Urinary neutrophil gelatinase-associated lipocalin to monitor lupus nephritis disease activity. biomark Insights. (2015) 10:81–7. doi: 10.4137/BMI.S27625 PMC4562555 26396491

[21] Khanna A Plummer M Bromberek C Bresnahan B Hariharan S. Expression of TGF-beta and fibrogenic genes in transplant recipients with tacrolimus and cyclosporine nephrotoxicity. Kidney Int. (2002) 62:2257–63. doi: 10.1046/j.1523-1755.2002.00668.x 12427154

[22] Eddy AA Giachelli CM. Renal expression of genes that promote interstitial inflammation and fibrosis in rats with protein-overload proteinuria. Kidney Int. (1995) 47:1546–57. doi: 10.1038/ki.1995.218 7643523

[23] González-Cuadrado S Bustos C Ruiz-Ortega M Ortiz A Guijarro C Plaza JJ. Expression of leucocyte chemoattractants by interstitial renal fibroblasts: up-regulation by drugs associated with interstitial fibrosis. Clin Exp Immunol. (1996) 106:518–22. doi: 10.1046/j.1365-2249.1996.d01-864.x PMC2200627 8973621

[24] Tesch GH Schwarting A Kinoshita K Lan HY Rollins BJ Kelley VR. Monocyte chemoattractant protein-1 promotes macrophage-mediated tubular injury, but not glomerular injury, in nephrotoxic serum nephritis. J Clin Invest. (1999) 103:73–80. doi: 10.1172/JCI4876 9884336 PMC407867

[25] Mishra J Ma Q Prada A Mitsnefes M Zahedi K Yang J. Identification of neutrophil gelatinase-associated lipocalin as a novel early urinary biomarker for ischemic renal injury. J Am Soc Nephrol. (2003) 14:2534–43. doi: 10.1097/01.asn.0000088027.54400.c6 14514731

[26] Frangogiannis N. Transforming growth factor-β in tissue fibrosis. J Exp Med. (2020) 217:e20190103. doi: 10.1084/jem.20190103 32997468 PMC7062524

[27] Han WK Bailly V Abichandani R Thadhani R Bonventre JV. Kidney Injury Molecule-1 (KIM-1): a novel biomarker for human renal proximal tubule injury. Kidney Int. (2002) 62:237–44. doi: 10.1046/j.1523-1755.2002.00433.x 12081583

[28] van Timmeren MM Vaidya VS van Ree RM Oterdoom LH de Vries AP Gans RO. High urinary excretion of kidney injury molecule-1 is an independent predictor of graft loss in renal transplant recipients. Transplantation. (2007) 84:1625–30. doi: 10.1097/01.tp.0000295982.78039.ef PMC2745062 18165774

[29] Nogare AL Veronese FV Carpio VN Montenegro RM Pedroso JA Pegas KL. Kidney injury molecule-1 expression in human kidney transplants with interstitial fibrosis and tubular atrophy. BMC Nephrol. (2015) 16:19. doi: 10.1186/s12882-015-0011-y 25884518 PMC4359521

[30] Shinke H Masuda S Togashi Y Ikemi Y Ozawa A Sato T. Urinary kidney injury molecule-1 and monocyte chemotactic protein-1 are noninvasive biomarkers of cisplatin-induced nephrotoxicity in lung cancer patients. Cancer Chemother Pharmacol. (2015) 76:989–96. doi: 10.1007/s00280-015-2880-y PMC4624288 26407820

[31] Wajda J Dumnicka P Kolber W Sporek M Maziarz B Ceranowicz P. The marker of tubular injury, kidney injury molecule-1 (KIM-1), in acute kidney injury complicating acute pancreatitis: A preliminary study. J Clin Med. (2020) 9(5):1463. doi: 10.3390/jcm9051463 32414176 PMC7290845

[32] Ghobrial EE El Hamshary AA Mohamed AG Abd El Raheim YA Talaat AA. Urinary monocyte chemoattractant protein-1 as a biomarker of lupus nephritis activity in children. Saudi J Kidney Dis Transpl. (2015) 26:507–15. 10.4103/1319-2442.157350 26022021

[33] Cantaluppi V Dellepiane S Tamagnone M Medica D Figliolini F Messina M. Neutrophil gelatinase associated lipocalin is an early and accurate biomarker of graft function and tissue regeneration in kidney transplantation from extended criteria donors. PloS One. (2015) 10:e0129279. doi: 10.1371/journal.pone.0129279 26125566 PMC4488380

[34] Nozaki Y Shiga T Ashida C Tomita D Itami T Kishimoto K. U-KIM-1 as a predictor of treatment response in lupus nephritis. Lupus. (2023) 32:54–62. doi: 10.1177/09612033221135871 36305170

[35] Ding Y Nie LM Pang Y Wu WJ Tan Y Yu F. Composite urinary biomarkers to predict pathological tubulointerstitial lesions in lupus nephritis. Lupus. (2018) 27:1778–89. doi: 10.1177/0961203318788167 30020021

[36] Brunner HI Bennett MR Abulaban K Klein-Gitelman MS O’Neil KM Tucker L. Development of a novel renal activity index of lupus nephritis in children and young adults. Arthritis Care Res (Hoboken). (2016) 68:1003–11. doi: 10.1002/acr.22762 PMC4834060 26473509

[37] Carlos CP Sonehara NM Oliani SM Burdmann EA. Predictive usefulness of urinary biomarkers for the identification of cyclosporine A-induced nephrotoxicity in a rat model. PloS One. (2014) 9:e103660. doi: 10.1371/journal.pone.0103660 25072153 PMC4114979

[38] Fu R Tajima S Shigematsu T Zhang M Tsuchimoto A Egashira N. Establishment of an experimental rat model of tacrolimus-induced kidney injury accompanied by interstitial fibrosis. Toxicol Lett. (2021) 341:43–50. doi: 10.1016/j.toxlet.2021.01.020 33516819

[39] Kędzierska K Sindrewicz K Sporniak-Tutak K Gołembiewska E Zair L Sieńko J. Does immunosuppressive therapy affect markers of kidney damage? Ann Transplant. (2016) 21:137–44. doi: 10.12659/aot.895275 26936590

[40] Tsuchimoto A Shinke H Uesugi M Kikuchi M Hashimoto E Sato T. Urinary neutrophil gelatinase-associated lipocalin: a useful biomarker for tacrolimus-induced acute kidney injury in liver transplant patients. PloS One. (2014) 9:e110527. doi: 10.1371/journal.pone.0110527 25329716 PMC4203804

[41] Stypmann J Fobker M Rosing K Engelen M Gunia S Dell’Aquila AM. Neutrophil gelatinase-associated lipocalin (NGAL) in heart transplant recipients after conversion to everolimus therapy. J Cardiol. (2015) 66:347–52. doi: 10.1016/j.jjcc.2014.12.010 25583090

[42] Gacka E Życzkowski M Bogacki R Paradysz A Hyla-Klekot L. The usefulness of determining neutrophil gelatinase-associated lipocalin concentration excreted in the urine in the evaluation of cyclosporine A nephrotoxicity in children with nephrotic syndrome. Dis Markers. (2016) 2016:6872149. doi: 10.1155/2016/6872149 28115789 PMC5220415

[43] Chancharoenthana W Leelahavanichkul A Wattanatorn S Avihingsanon Y Praditpornsilpa K Eiam-Ong S. Alteration of urinary neutrophil gelatinase-associated lipocalin as a predictor of tacrolimus-induced chronic renal allograft fibrosis in tacrolimus dose adjustments following kidney transplantation. PloS One. (2018) 13:e0209708. doi: 10.1371/journal.pone.0209708 30576367 PMC6303063

[44] Mohamed MA Robertson H Booth TA Balupuri S Gerstenkorn C Kirby JA. Active TGF-beta1 expression in kidney transplantation: a comparative study of cyclosporin-A (CyA) and tacrolimus (FK506). Transpl Int. (2000) 13 Suppl 1:S295–8. doi: 10.1007/s001470050346 11112017

[45] Mohamed MA Robertson H Booth TA Balupuri S Kirby JA Talbot D. TGF-beta expression in renal transplant biopsies: a comparative study between cyclosporin-A and tacrolimus. Transplantation. (2000) 69:1002–5. doi: 10.1097/00007890-200003150-00059. 10755567

[46] Matl I Viklický O Voska L Lodererová A Vítko S. The effect of different immunosuppressive regimens on TGF-beta1 expression in kidney transplant patients. Transpl Int. (2005) 18:668–71. doi: 10.1111/j.1432-2277.2005.00115.x 15910291

[47] Citterio F Pozzetto U Romagnoli J Tondolo E Silvestri P Nanni G. Plasma levels of transforming growth factor-beta1 in renal transplant recipients receiving different immunosuppressive regimens. Transplant Proc. (2004) 36:698–9. doi: 10.1016/j.transproceed.2004.03.014 15110635

[48] Khanna A Plummer M Bromberek C Bresnahan B Hariharan S. Expression of TGF-β and fibrogenic genes in transplant recipients with tacrolimus and cyclosporine nephrotoxicity. Kidney Int. (2002) 62:2257–63. doi: 10.1046/j.1523-1755.2002.00668.x 12427154

[49] Ozdemir BH Ozdemir FN Demirhan B Haberal M. TGF-beta1 expression in renal allograft rejection and cyclosporine A toxicity. Transplantation. (2005) 80:1681–5. doi: 10.1097/01.tp.0000185303.92981.d6 16378061

[50] Roos-van-Groningen MC Scholten EM Lelieveld PM Rowshani AT Baelde HJ Bajema IM. Molecular comparison of calcineurin inhibitor-induced fibrogenic responses in protocol renal transplant biopsies. J Am Soc Nephrol. (2006) 17:881–8. doi: 10.1681/ASN.2005080891 16467444

[51] Boswell JM Yui MA Burt DW Kelley VE. Increased tumor necrosis factor and IL-1 beta gene expression in the kidneys of mice with lupus nephritis. J Immunol. (1988) 141:3050–4. doi: 10.4049/jimmunol.141.9.3050 3262676

[52] Timoshanko JR Kitching AR Iwakura Y Holdsworth SR Tipping PG. Contributions of IL-1beta and IL-1alpha to crescentic glomerulonephritis in mice. J Am Soc Nephrol. (2004) 15:910–8. doi: 10.1097/01.asn.0000115704.86897.f4 15034093

[53] Bilsborrow JB Doherty E Tilstam PV Bucala R. Macrophage migration inhibitory factor (MIF) as a therapeutic target for rheumatoid arthritis and systemic lupus erythematosus. Expert Opin Ther Targets. (2019) 23:733–44. doi: 10.1080/14728222.2019.1656718 PMC6800059 31414920

[54] Gamez-Nava JI Diaz-Rizo V Perez-Guerrero EE Muñoz-Valle JF Saldaña-Cruz AM Fajardo-Robledo NS. Assessment of serum macrophage migration inhibitory factor (MIF), adiponectin, and other adipokines as potential markers of proteinuria and renal dysfunction in lupus nephritis: a cross-sectional study. biomark Res. (2020) 8:55. doi: 10.1186/s40364-020-00236-x 33133605 PMC7594329

[55] Lee HT Shiao YM Wu TH Chen WS Hsu YH Tsai SF. Serum BLC/CXCL13 concentrations and renal expression of CXCL13/CXCR5 in patients with systemic lupus erythematosus and lupus nephritis. J Rheumatol. (2010) 37:45–52. doi: 10.3899/jrheum.090450 19955043

[56] Arriens C Teng YKO Ginzler EM Parikh SV Askanase AD Saxena A. Update on the efficacy and safety profile of voclosporin: an integrated analysis of clinical trials in lupus nephritis. Arthritis Care Res. (2022) 75(7):1399–408. doi: 10.1002/acr.25007 36039949

[57] Kamel KS Ethier JH Quaggin S Levin A Albert S Carlisle EJ. Studies to determine the basis for hyperkalemia in recipients of a renal transplant who are treated with cyclosporine. J Am Soc Nephrol. (1992) 2(8):1279–84. doi: 10.1681/ASN.V281279 1627752

[58] Nawaz SH Zafar MN Naqvi SA Rizvi SA. Impact of cyclosporin immunosuppression on serum magnesium and its fractional excretion in renal transplant recipients. J Pak Med Assoc. (2005) 55(3):98–100. 15852743

[59] Mazzola BL Vannini SD Truttmann AC von Vigier RO Wermuth B Ferrari P. Long-term calcineurin inhibition and magnesium balance after renal transplantation. Transpl Int. (2003) 16(2):76–81. doi: 10.1007/s00147-002-0479-9 12595968

[60] Gratreak BDK Swanson EA Lazelle RA Jelen SK Hoenderop J Bindels RJ. Tacrolimus-induced hypomagnesemia and hypercalciuria requires FKBP12 suggesting a role for calcineurin. Physiol Rep. (2020) 8(8):e14316. doi: 10.14814/phy2.14316 31908154 PMC6944708

