# Supplementary material for: The kidney injury biomarker profile of patients with lupus nephritis remains unchanged with the second-generation calcineurin inhibitor voclosporin
Source: Front Nephrol. 2025 Mar 17;5:1540471. doi: 10.3389/fneph.2025.1540471 (PMC11955810; doi:10.3389/fneph.2025.1540471)
Supplement: Supplementary file 1 [file DataSheet1.docx]

Supplementary Material

Supplemental Table 1. Inclusion and exclusion criteria in the AURORA 1 study

| **Inclusion criteria** |
| --- |
| 1. Written informed consent before any study-specific procedures are performed. 2. Male or female patients with a minimum age of 18 (or legal age of consent if >18 years) to 75 years of age, inclusive, at the time of screening. 3. Previous diagnosis of systemic lupus erythematosus (SLE) according to the American College of Rheumatology (ACR) criteria (1997). 4. Patients with evidence of active lupus nephritis (LN), defined as follows:  - Kidney biopsy result within 2 years prior to screening indicating Class III, IV-S, IV-G (alone or in combination with Class V), or Class V LN with a doubling or greater increase of urine protein creatinine ratio (UPCR) within the last 6 months to a minimum of ≥1.5 mg/mg for Class III/IV or to a minimum of ≥2 mg/mg for Class V at screening. Biopsy results over 6 months prior to screening must be reviewed with a medical monitor to confirm eligibility.   OR   - Kidney biopsy result within 6 months prior to screening indicating Class III, IV-S, or IV-G (alone or in combination with Class V) LN with a UPCR of ≥1.5 mg/mg at screening.   OR   - Kidney biopsy result within 6 months prior to screening indicating Class V LN and a UPCR of ≥2 mg/mg at screening.   A biopsy can be performed during screening, if not available. The above criteria must be fulfilled at baseline.   1. In the opinion of the Investigator, patient requires high-dose corticosteroids and immunosuppressive therapy. 2. Patient is willing to take oral mycophenolate mofetil (MMF) for the duration of the study, either by continuing current MMF therapy or by initiating it on or before the baseline visit. 3. Women of childbearing potential must have a negative serum pregnancy test at screening and a negative urine pregnancy test at baseline. Two effective forms of contraception must be used simultaneously unless abstinence is the chosen method. Patients must use effective contraception during the study. |
| **Exclusion criteria** |
| 1. Patients unable or unwilling to give written informed consent and/or to comply with study procedures. 2. Estimated glomerular filtration rate (eGFR) as calculated by the Chronic Kidney Disease Epidemiology Collaboration (CKD-EPI) equation of ≤45 mL/min/1.73 m^2^ at screening confirmed before randomization. 3. Currently taking or known need for any of the medications prohibited in the study protocol; this includes prohibited medications prior to screening. 4. Currently requiring renal dialysis (hemodialysis or peritoneal dialysis) or expected to require dialysis during the study period. 5. A previous kidney transplant or planned transplant within study treatment period. 6. Any known hypersensitivity or contraindication to MMF, mycophenolic acid, cyclosporine, corticosteroids, or any components of these drug products. 7. Current or medical history of:  - Congenital or acquired immunodeficiency. - In the opinion of the Investigator, clinically significant drug, or alcohol abuse within 2 years prior to screening. - Malignancy within 5 years of screening, with the exception of basal and squamous cell carcinomas treated by complete excision. Patients with cervical dysplasia that is cervical intraepithelial neoplasia 1 but have been treated with conization or loop electrosurgical excision procedure and have had a normal repeat Papanicolaou test are allowed. - Lymphoproliferative disease or previous total lymphoid irradiation. - Severe viral infection (e.g., cytomegalovirus, hepatitis B virus, hepatitis C virus) within 3 months of screening; or known HIV infection. Severe viral infection is defined as active disease requiring antiviral therapy. - Active tuberculosis (TB) or known history of TB or evidence of old TB if not taking prophylaxis with isoniazid.  1. Other known clinically significant active medical conditions, such as:  - Severe cardiovascular disease including congestive heart failure, history of cardiac dysrhythmia or congenital long QT syndrome. QT interval duration corrected for heart rate using method of Fridericia (QTcF) exceeding 480 msec in the presence of a normal QRS interval (<110 msec) at time of screening will result in exclusion. - Liver dysfunction (aspartate aminotransferase, alanine aminotransferase, or bilirubin ≥2.5 times the upper limit of normal) at screening and, if abnormal at screening, then confirmed that the levels have returned to <2.5 times upper limit of normal before randomization. - Chronic obstructive pulmonary disease or asthma requiring oral steroids. - Bone marrow insufficiency unrelated to active SLE (according to Investigator judgment) with white blood cell count <2,500/mm3; absolute neutrophil count (ANC) <1.3×10^3^/μL; thrombocytopenia (platelet count <50,000/mm^3^). - Active bleeding disorders. - Current infection requiring intravenous antibiotics.  1. Any overlapping autoimmune condition for which the condition or the treatment of the condition may affect the study assessments or outcomes (e.g., scleroderma with significant pulmonary hypertension; any condition for which additional immunosuppression is indicated). Overlapping conditions for which the condition or treatment is not expected to affect assessments or outcomes (e.g., Sjögren’s syndrome) are not excluded. 2. No vaccines using live organisms, virus or bacterial, are allowed during screening and while taking the study treatment. 3. Other major physical or psychiatric illness or major traumatic injury within 6 months prior to screening that may affect study conduct or interfere with study assessments or outcome. 4. Any other medical condition which, in the Investigator’s judgment, may be associated with increased risk to the patient or may interfere with study assessments or outcomes. 5. Patients who are pregnant, breast feeding or, if of childbearing potential, not using adequate contraceptive precautions. 6. Participation in another clinical study within 4 weeks prior to screening and/or receipt of investigational drugs within 4 weeks or 5 half-lives of the drug (whichever is longer) prior to screening. 7. Patients randomized and treated in a previous voclosporin clinical study. |

**Supplemental Table 2**. Urinary and serum biomarkers analyzed in samples from patients with active lupus nephritis. ^a^Of the 43 urinary analytes assayed, 27 had consistently measurable levels. ^b^Of the 30 serum biomarkers assayed, 27 had consistently measurable levels.

| **Urinary biomarkers^a^** | **Serum biomarkers^b^** | **Urinary and serum biomarkers** |
| --- | --- | --- |
| Eotaxin-2 | Angiopoietin-1 | Brain-derived neurotrophic factor |
| Granulocyte-macrophage colony-stimulating factor | Angiopoietin-2 | Calbindin |
| Growth-regulated alpha protein | B-lymphocyte chemoattractant | Eotaxin-1 |
| Interferon gamma | Carbonic anhydrase 9 | Factor VII |
| Interleukin-1 receptor antagonist | Decorin | Intercellular adhesion molecule 1 |
| Interleukin-2 receptor alpha | Eotaxin-3 | Interleukin-1 alpha |
| Interleukin-2 | Insulin-like growth factor-binding protein 2 | Interleukin-1 beta |
| Interleukin-3 | Interleukin-1 receptor antagonist | Interleukin-12 subunit p70 |
| Interleukin-4 | Interleukin-12 subunit p40 | Interleukin-17 |
| Interleukin-5 | Interleukin-18-binding protein | Interleukin-23 |
| Interleukin-6 | Macrophage migration inhibitory factor | Kidney injury molecule-1 |
| Interleukin-7 | Platelet endothelial cell adhesion molecule | Matrix metalloproteinase-3 |
| Interleukin-8 | Pulmonary surfactant-associated protein D | Matrix metalloproteinase-9 |
| Interleukin-10 |  | Osteopontin |
| Interleukin-12 subunit p40 |  | Stem cell factor |
| Interleukin-18 |  | Tamm-Horsfall urinary glycoprotein |
| Latency-associated peptide of transforming growth factor beta 1 |  | Vascular endothelial growth factor |
| Macrophage inflammatory protein-1 alpha |  |  |
| Macrophage inflammatory protein-1 beta |  |  |
| Monocyte chemotactic protein 1 |  |  |
| Neutrophil gelatinase-associated lipocalin |  |  |
| Osteoprotegerin |  |  |
| TrefoilfFactor 3 |  |  |
| Tumor necrosis factor alpha |  |  |
| Tumor necrosis factor beta |  |  |
| Tumor necrosis factor receptor 1 |  |  |

**Supplemental Table 3**. **Changes in the normalized levels of urinary analytes from baseline to EoT in all-patient and eGFR decrease cohorts.** Analysis of patients randomly selected from each treatment arm of AURORA 1, including patients who experienced a ≥30% decline from baseline in eGFR during the study. Data is presented as the CFB to EoT of LS means (SE) of normalized levels of urinary analytes. Also presented are estimates of the difference in CFB (95% CI) between the voclosporin and control arms. Significant changes in the levels of analytes from baseline to EoT are highlighted in bold. ^a^eGFR decrease defined as ≥30% reduction in eGFR from baseline during the study. ^b^Median (IQR) treatment duration for eGFR decrease cohort was 22.9 (15.8 – 37.4) weeks for placebo-treated patients and 26.2 (16.7 – 51.1) weeks for voclosporin-treated patients. For all other patients included in this post hoc analysis the median (IQR) treatment duration was 52 (50.86, 53.14) weeks for placebo-treated patients and 52 (51.14, 53.14) weeks for voclosporin-treated patients CFB, change from baseline; CI, confidence interval; eGFR, estimated glomerular filtration rate; EoT, end of treatment; IQR, interquartile range; LS, least squares; SE, standard error.

| **Analyte** | **All-patient cohort** | | | **eGFR decrease cohort^a^** | | |
| --- | --- | --- | --- | --- | --- | --- |
|  | **CFB to EoT^b^, LS means (SE)** | | | | | |
|  | **Voclosporin (n=25)** | **Control (n=25)** | **Estimate CFB difference vs control (95% CI)** | **Voclosporin (n=13)** | **Control (n=8)** | **Estimate CFB difference vs control (95% CI)** |
| Calbindin | -1.7 (0.29) | -1.4 (0.29) | -0.3 (-1.1, 0.5) | -1.7 (0.46) | -1.0 (0.59) | -0.8 (-2.4, 0.8) |
| Eotaxin-1 | **-0.4 (0.12)** | **0.1 (0.12)** | **-0.5 (-0.8, -0.1)** | **-0.4 (0.13)** | **0.4 (0.16)** | **-0.8 (-1.2, -0.3)** |
| Eotaxin-2 | -0.1 (0.03) | -0.1 (0.03) | 0.0 (-0.1, 0.1) | -0.1 (0.05) | -0.0 (0.07) | -0.1 (-0.2, 0.1) |
| Factor VII | 0.02 (0.026) | -0.02 (0.026) | 0.03 (-0.04, 0.10) | 0.05 (0.049) | 0.03 (0.063) | 0.02 (-0.15, 0.19) |
| Growth-regulated alpha protein | -0.05 (0.078) | -0.16 (0.078) | 0.11 (-0.11, 0.33) | 0.11 (0.089) | 0.02 (0.113) | 0.10 (-0.21, 0.40) |
| Intercellular adhesion molecule 1 | -0.04 (0.011) | -0.03 (0.011) | -0.02 (-0.05, 0.02) | -0.06 (0.013) | -0.05 (0.017) | -0.01 (-0.06, 0.03) |
| Interleukin-6 | -0.07 (0.018) | -0.07 (0.018) | -0.00 (-0.05, 0.05) | -0.04 (0.026) | 0.02 (0.034) | -0.06 (-0.15, 0.03) |
| Interleukin-8 | -0.6 (0.38) | -0.7 (0.38) | 0.1 (-1.0, 1.2) | -0.7 (0.60) | 0.5 (0.76) | -1.2 (-3.3, 0.8) |
| Interleukin-17 | -0.007 (0.0034) | 0.002 (0.0034) | -0.008 (-0.018, 0.001) | -0.012 (0.0037) | -0.003 (0.0048) | -0.010 (-0.022, 0.003) |
| Interleukin-1 alpha | **-0.01 (0.004)** | **0.01 (0.004)** | **-0.02 (-0.03, -0.00)** | -0.02 (0.007) | 0.00 (0.008) | -0.02 (-0.04, 0.00) |
| Interleukin-1 beta | **-0.01 (0.008)** | **0.01 (0.008)** | **-0.03 (-0.05, -0.00)** | **-0.02 (0.013)** | **0.03 (0.016)** | **-0.05 (-0.09, -0.01)** |
| Interleukin-1 receptor antagonist | 0.3 (2.20) | 0.0 (2.20) | 0.2 (-6.0, 6.5) | -5.4 (3.62) | -6.0 (4.65) | 0.6 (-12.0, 13.1) |
| Interleukin-2 receptor alpha | -13.4 (4.27) | -11.1 (4.27) | -2.3 (-14.5, 9.9) | -20.6 (4.29) | -17.2 (5.49) | -3.4 (-18.1, 11.4) |
| Interleukin-12 subunit p40 | -0.00078 (0.000643) | 0.00068 (0.000643) | -0.00146  (-0.00333, 0.00040) | -0.00140 (0.000587) | -0.00041 (0.000756) | -0.00099  (-0.00304, 0.00106) |
| Kidney injury molecule-1 | -0.008  (0.0032) | -0.007  (0.0032) | -0.002  (-0.011, 0.008) | -0.006  (0.0060) | 0.004  (0.0077) | -0.010  (-0.031, 0.011) |
| Latency-associated peptide of transforming growth factor beta 1 | -0.0018  (0.00029) | -0.0016 (0.00029) | -0.0002  (-0.0010, 0.0007) | -0.0021  (0.00051) | -0.0009 (0.00065) | -0.0013  (-0.0030, 0.0005) |
| Macrophage inflammatory protein-1 beta | -0.6 (0.21) | -0.6 (0.21) | -0.0 (-0.6, 0.6) | -0.9 (0.38) | -0.4 (0.48) | -0.5 (-1.8, 0.7) |
| Matrix metalloproteinase-9 | -0.09 (0.049) | 0.03 (0.049) | -0.12 (-0.26, 0.02) | -0.12 (0.060) | 0.02 (0.077) | -0.14 (-0.35, 0.06) |
| Monocyte chemotactic protein 1 | -15.0 (3.30) | -19.1 (3.30) | 4.1 (-5.3, 13.5) | -12.0 (3.98) | -1.5 (5.09) | -10.5 (-24.1, 3.1) |
| Neutrophil gelatinase-associated lipocalin | -0.0 (0.89) | -1.1 (0.89) | 1.0 (-1.5, 3.6) | 1.4 (1.84) | -0.1 (2.34) | 1.5 (-4.8, 7.7) |
| Osteopontin | -0.4 (2.28) | -1.7 (2.28) | 1.3 (-5.2, 7.8) | -4.2 (2.59) | -2.6 (3.30) | -1.6 (-10.4, 7.2) |
| Osteoprotegerin | -0.0262 (0.01195) | -0.0270 (0.01195) | 0.0008  (-0.0334, 0.0350) | -0.0179 (0.01900) | 0.0431 (0.02437) | -0.0610  (-0.1267, 0.0048) |
| Stem cell factor | -2.7 (0.59) | -1.5 (0.59) | -1.2 (-2.9, 0.4) | -3.9 (0.83) | -1.9 (1.06) | -2.0 (-4.9, 0.8) |
| Tamm-Horsfall urinary glycoprotein | -0.03 (0.014) | 0.01 (0.014) | -0.04 (-0.08, 0.00) | -0.04 (0.011) | -0.00 (0.014) | -0.03 (-0.07, 0.00) |
| Trefoil factor 3 | 0.028 (0.0146) | -0.008 (0.0146) | 0.036 (-0.005, 0.078) | 0.026 (0.0193) | -0.001 (0.0248) | 0.027 (-0.040, 0.094) |
| Tumor necrosis factor receptor I | -0.9 (13.89) | -23.0 (13.89) | 22.0 (-17.5, 61.6) | 12.4 (23.86) | 15.3 (30.51) | -2.8 (-84.7, 79.0) |
| Vascular endothelial growth factor | -0.9 (0.40) | -0.9 (0.40) | -0.1 (-1.2, 1.1) | -0.4 (0.75) | -0.5 (0.96) | 0.1 (-2.5, 2.6) |

**Supplemental Table 4**. **Changes in the absolute levels of urinary analytes from baseline to EoT in all-patient and eGFR decrease cohorts.** Analysis of patients randomly selected from each treatment arm of AURORA 1, including patients who experienced a ≥30% decline from baseline in eGFR during the study. Data is presented as the CFB to EoT of LS means (SE) of absolute levels of urinary analytes. Also presented are estimates of the difference in CFB (95% CI) between the voclosporin and control arms. Significant changes in the levels of analytes from baseline to EoT are highlighted in bold. ^a^eGFR decrease defined as ≥30% reduction from baseline in eGFR during the study. ^b^Median (IQR) treatment duration for eGFR decrease cohort was 22.9 (15.8 – 37.4) weeks for placebo-treated patients and 26.2 (16.7 – 51.1) weeks for voclosporin-treated patients. For all other patients included in this post hoc analysis the median (IQR) treatment duration was 52 (50.86, 53.14) weeks for placebo-treated patients and 52 (51.14, 53.14) weeks for voclosporin-treated patients CFB, change from baseline; CI, confidence interval; eGFR, estimated glomerular filtration rate; EoT, end of treatment; IQR, interquartile range; LS, least squares; SE, standard error.

| **Analyte** | **All-patient cohort** | | | **eGFR decrease cohort^a^** | | |
| --- | --- | --- | --- | --- | --- | --- |
|  | **CFB to EoT^b^, LS means (SE)** | | | | | |
|  | **Voclosporin (n=25)** | **Control (n=25)** | **Estimate CFB difference vs control (95% CI)** | **Voclosporin (n=13)** | **Control (n=8)** | **Estimate CFB difference vs control (95% CI)** |
| Calbindin | -129.7 (18.68) | -138.0 (18.68) | 8.2 (-44.9, 61.4) | -100.2 (22.09) | -120.9 (28.31) | 20.7 (-55.5, 96.9) |
| Eotaxin-1 | -16.1 (7.29) | 3.2 (7.29) | -19.3 (-40.7, 2.2) | -9.2 (11.58) | 18.8 (14.93) | -28.0 (-68.6, 12.6) |
| Eotaxin-2 | 5.7 (4.88) | -8.0 (4.88) | 13.6 (-0.3, 27.5) | 14.4 (9.71) | -5.4 (12.38) | 19.8 (-13.2, 52.9) |
| Factor VII | 11.45 (4.544) | -1.28 (4.544) | 12.72 (-0.24, 25.68) | 17.91 (9.476) | 0.98 (12.108) | 16.94 (-15.52, 49.39) |
| Growth-regulated alpha protein | **13.45 (9.163)** | **-14.88 (9.163)** | **28.33 (2.19, 54.48)** | 38.08 (14.445) | -6.55 (18.415) | 44.63 (-4.55, 93.80) |
| Intercellular adhesion molecule 1 | -1.96 (0.809) | -1.87 (0.809) | -0.10 (-2.40, 2.21) | -2.05 (0.982) | -2.86 (1.254) | 0.81 (-2.55, 4.17) |
| Interleukin-6 | 112.1 (517.96) | -708.1 (517.96) | 820.2 (-662.2, 2302.7) | 0.72 (2.427) | -0.35 (3.096) | 1.06 (-7.21, 9.34) |
| Interleukin-8 | **-7.1 (25.04)** | **-83.7 (25.04)** | **76.6 (5.2, 148.0)** | 8.5 (34.63) | -24.9 (44.32) | 33.4 (-85.7, 152.4) |
| Interleukin-17 | -0.101 (0.2112) | 0.135 (0.2112) | -0.236 (-0.853, 0.382) | -0.294 (0.2334) | -0.336 (0.3009) | 0.072 (-0.746, 0.890) |
| Interleukin-1 alpha | -0.18 (0.259) | 0.34 (0.259) | -0.52 (-1.26, 0.22) | -0.80 (0.318) | -0.75 (0.408) | -0.05 (-1.15, 1.05) |
| Interleukin-1 beta | 0.30 (0.530) | 0.41 (0.530) | -0.11 (-1.62, 1.40) | 0.46 (0.927) | 0.34 (1.185) | 0.11 (-3.06, 3.29) |
| Interleukin-1 receptor antagonist | 691.6 (579.90) | -232.6 (579.90) | 924.2 (-729.1, 2577.5) | 726.4 (1252.70 | -1010.4 (1609.28) | 1746.8 (-2602.7, 6096.4) |
| Interleukin-2 receptor alpha | 112.1 (517.96) | -708.1 (517.96) | 820.2 (-662.2, 2302.7) | 48.3 (714.70) | -979.5 (921.80) | 1027.8 (-1478.8, 3534.3) |
| Interleukin-12 subunit p40 | -0.01426 (0.039550) | 0.06004 (0.039550) | -0.07431 (-018965, 0.04104) | -0.02470 (0.039917) | -0.04929 (0.051559) | 0.02459 (-0.11592, 0.16510) |
| Kidney injury molecule-1 | -0.402 (0.3244) | -0.392 (0.3244) | -0.010 (-0.938, 0.918) | 0.119 (0.6630) | 0.713 (0.8452) | -0.595 (-2.852, 1.662) |
| Latency-associated peptide of transforming growth factor beta 1 | -0.0459 (0.03083) | -0.1161 (0.03083) | 0.0702 (-0.0191, 0.1594) | -0.0147 (0.06003) | -0.0878 (0.07667) | 0.0731 (-0.1323, 0.2785) |
| Macrophage inflammatory protein-1 beta | -16.4 (17.24) | -64.9 (17.24) | 48.5 (-0.7, 97.6) | -15.3 (31.79) | -69.1 (40.67) | 53.8 (-55.4, 163.0) |
| Matrix metalloproteinase-9 | -1.60 (3.112) | -1.46 (3.112) | -0.13 (-8.99, 8.72) | -0.26 (4.556) | -4.83 (5.835) | 4.57 (-11.13, 20.26) |
| Monocyte chemotactic protein 1 | **-411.9 (355.04)** | **-1458.5 (355.04)** | **1046.6 (35.0, 2058.2)** | 567.8 (474.33) | -508.3 (604.98) | 1076.1 (-540.7, 2693.0) |
| Neutrophil gelatinase-associated lipocalin | 164.5 (111.11) | -75.9 (111.11) | 240.3 (-78.2, 558.8) | 366.2 (236.30) | -22.2 (301.50) | 388.3 (-417.9, 1194.6) |
| Osteopontin | 330.2 (215.74) | -201.2 (215.74) | 531.4 (-82.8, 1145.5) | 124.4 (324.29) | -227.9 (413.53) | 352.3 (-752.5, 1457.1) |
| Osteoprotegerin | 0.6042 (1.15212) | -2.5676 (1.15212) | 3.1718 (-0.1242, 6.4678) | 2.5912 (2.20445) | 0.3568 (2.83301) | 2,2345 (-5.4271, 9.8960) |
| Stem cell factor | -138.0 (42.37) | -119.8 (42.37) | -18.2 (-139.1, 102.6) | -177.9 (41.91) | -156.5 (53.74) | -21.4 (-166.2, 123.4) |
| Tamm-Horsfall urinary glycoprotein | -0.64 (0.694) | 0.69 (0.694) | -1.33 (-3.31, 0.65) | -0.36 (0.998) | -0.96 (1.276) | 0.60 (02.82, 4.02) |
| Trefoil factor 3 | **5.227 (1.8347)** | **-0.639 (1.8347)** | **5.865 (0.644, 11.087)** | 6.623 (3.1617) | -0.500 (4.0333) | 7.123 (-3.659, 17.905) |
| Tumor necrosis factor receptor I | **4334.3 (2020.73)** | **-2170.8 (2020.73)** | **6505.2 (750.0, 12260.4)** | 7689.1 (4127.42) | -903.4 (5283.27) | 8592.6 (-5607.7, 22792.8) |
| Vascular endothelial growth factor | -93.7 (67.67) | -92.7 (67.67) | 186.4 (-7.2, 380.1) | 257.1 (136.25) | -121.7 (174.72) | 378.8 (-92.1, 849.7) |

**Supplemental Table 5**. **Changes in serum analyte levels from baseline to EoT in all-patient and eGFR decrease cohorts.** Analysis of patients randomly selected from each treatment arm of AURORA 1 including patients who experienced a ≥30% decline in eGFR from baseline during the study. Data is presented as CFB to EoT of LS means (SE) of levels of serum analytes. Also presented are estimates of the difference in CFB (95% CI) between the voclosporin and control arms. Significant changes in the levels of analytes from baseline to EoT are highlighted in bold. ^a^eGFR decrease defined as ≥30% reduction from baseline in eGFR during the study. ^b^Median (IQR) treatment duration for eGFR decrease cohort was 22.9 (15.8 – 37.4) weeks for placebo-treated patients and 26.2 (16.7 – 51.1) weeks for voclosporin-treated patients. For all other patients included in this post hoc analysis the median (IQR) treatment duration was 52 (50.86, 53.14) weeks for placebo-treated patients and 52 (51.14, 53.14) weeks for voclosporin-treated patients CFB, change from baseline; CI, confidence interval; eGFR, estimated glomerular filtration rate; EoT, end of treatment; IQR, interquartile range; LS, least squares; SE, standard error.

| **Analyte (Unit of Measurement)** | **All-patient cohort** | | | **eGFR decrease cohort^a^** | | |
| --- | --- | --- | --- | --- | --- | --- |
| **CFB to EoT^b^, LS means (SE)** | | | | | | |
|  | **Voclosporin (n=53)** | **Control (n=54)** | **Estimate CFB difference vs control (95% CI)** | **Voclosporin (n=23)** | **Control (n=19)** | **Estimate CFB difference vs control (95% CI)** |
| Angiopoietin-1 (ng/mL) | -4.0 (1.87) | -5.5 (1.85) | 1.5 (-3.7, 6.7) | -5.2 (3.12) | -7.8 (3.43) | 2.6 (-6.8, 12.0) |
| Angiopoietin-2 (ng/mL) | 1.75 (1.231) | -1.34 (1.220) | 3.09 (-0.35, 6.54) | 4.77 (2.697) | -0.07 (2.970) | 4.84 (-3.30, 12.98) |
| B lymphocyte chemoattractant (pg/mL) | 3.04 (27.092) | -56.83 (26.837) | 59.87 (-16.07, 135.82) | **6.38 (24.537)** | **-113.99 (27.266)** | **120.37 (42.43, 198.30)** |
| Brain-derived neurotrophic factor (ng/mL) | 3.29 (0.865) | 3.35 (0.857) | -0.07 (-2.48, 2.35) | 0.84 (1.367) | 0.66 (1.504) | 0.18 (-3.93, 4.29) |
| Calbindin (ng/mL) | 0.66 (0.800) | 0.78 (0.792) | -0.11 (-2.35, 2.13) | 2.08 (1.110) | 1.45 (1.222) | 0.63 (-2.73, 3.99) |
| Carbonic anhydrase 9 (ng/mL) | -0.015 (0.0122) | -0.029 (0.0121) | 0.014 (-0.020, 0.048) | -0.012 (0.0205) | -0.060 (0.0226) | 0.048 (-0.015, 0.110) |
| Decorin (ng/mL) | -0.19 (0.069) | -0.22 (0.069) | 0.03 (-0.16, 0.23) | 0.08 (0.105) | -0.14 (0.115) | 0.21 (-0.10, 0.53) |
| Eotaxin-1 (pg/mL) | -63.6 (16.01) | -55.8 (15.86) | -7.7 (-52.4, 37.0) | -55.9 (27.86) | -44.5 (30.67) | -11.4 (-95.4, 72.6) |
| Factor VII (ng/mL) | 8.9 (15.85) | 12.6 (15.70) | -3.7 (-48.0, 40.7) | 29.1 (23.64) | 54.8 (26.02) | -25.7 (-96.9, 45.6) |
| Insulin-like growth factor-binding protein 2 (ng/mL) | -29.4 (14.96) | -26.6 (14.82) | -2.8 (-44.5, 39.0) | 18.1 (28.07) | 16.3 (30.89) | 1.8 (-82.7, 86.3) |
| Intercellular adhesion molecule 1 (ng/mL) | 18.2 (10.25) | 9.7 (10.15) | 8.5 (-20.2, 37.2) | 32.3 (22.50) | 18.6 (24.76) | 13.7 (-54.0, 81.4) |
| Interleukin-1 beta (pg/mL) | -5.92 (0.556) | -5.93 (0.551) | 0.01 (-1.54, 1.57) | -13.82 (0.851) | -13.53 (0.938) | -0.28 (-2.86, 2.29) |
| Interleukin-1 receptor antagonist (pg/mL) | -10.3 (14.52) | -23.2 (14.39) | 12.9 (-27.8, 53.5) | -33.0 (23.56) | -42.8 (25.97) | 9.9 (-61.7, 81.5) |
| Interleukin-12 subunit p40 (ng/mL) | 0.004 (0.0462) | 0.005 (0.0458) | -0.001 (-0.130, 0.128) | 0.035 (0.0698) | 0.095 (0.0771) | -0.060 (-0.274, 0.153) |
| Interleukin-17 (pg/mL) | 0.02 (0.227) | 0.14 (0.225) | -0.12 (-0.75, 0.52) | -0.06 (0.364) | 0.18 (0.402) | -0.24 (-1.36, 0.88) |
| Interleukin-18-binding protein (ng/mL) | -0.00 (1.014) | -0.22 (1.004) | 0.22 (-2.61, 3.05) | 3.76 (1.870) | 2.69 (2.057) | 1.07 (-4.56, 6.69) |
| Interleukin-23 (ng/mL) | -0.24 (0214) | -0.25 (0.212) | 0.00 (-0.59, 0.60) | -0.17 (0.377) | 0.32 (0.415) | -0.49 (-1.63, 0.65) |
| Kidney injury molecule-1 (ng/mL) | -0.084 (0.0427) | -0.079 (0.0423) | -0.005 (-0.124, 0.114) | 0.092 (0.0801) | 0.087 (0.0882) | 0.005 (-0.237, 0.246) |
| Macrophage migration inhibitory factor (ng/mL) | **-0.350 (0.0493)** | **-0.534 (0.0488)** | **0.184 (0.046, 0.322)** | -0.346 (0.0849) | -0.536 (0.0935) | 0.190 (-0.067, 0.447) |
| Matrix metalloproteinase-3 (ng/mL) | -21.3 (3.77) | -25.6 (3.73) | 4.3 (-6.3, 14.8) | -14.2 (7.19) | -15.8 (7.95) | 1.6 (-20.6, 23.8) |
| Matrix metalloproteinase-9 (ng/mL) | -3.3 (2.64) | -4.3 (2.62) | 1.0 (-6.4, 8.4) | -3.2 (3.91) | 1.2 (4.31) | -4.4 (-16.2, 7.5) |
| Osteopontin (ng/mL) | -4.4 (1.72) | -3.1 (1.71) | -1.3 (-6.1, 3.5) | -1.2 (3.13) | 2.7 (3.46) | -3.8 (-13.4, 5.7) |
| Platelet endothelial cell adhesion molecule (ng/mL) | -7.7 (1.77) | -5.5 (1.75) | -2.2 (-7.1, 2.7) | -10.0 (3.16) | -5.3 (3.48) | -4.6 (-14.2, 4.9) |
| Pulmonary surfactant-associated protein D (ng/mL) | -0.65 (0.248) | -0.33 (0.246) | -0.32 (-1.01, 0.38) | -0.47 (0.341) | -0.65 (0.376) | 0.18 (-0.86, 1.22) |
| Stem cell factor (pg/mL) | 16.5 (60.40) | -49.6 (59.84) | 66.1 (-102.6, 234.8) | 309.2 (106.50) | 116.8 (117.38) | 192.4 (-131.1, 515.9) |
| Tamm-Horsfall urinary glycoprotein (µg/mL) | -0.009 (0.0019) | -0.005 (0.0019) | -0.004 (-0.010, 0.001) | -0.007 (0.0027) | -0.009 (0.0030) | 0.001 (-0.007, 0.010) |
| Vascular endothelial growth factor (pg/mL) | -30.9 (14.70) | -32.4 (14.56) | 1.5 (-39.6, 42.6) | -27.8 (27.14) | -35.1 (29.91) | 7.4 (-74.9, 89.7) |

**Supplemental Table 6.** **Fractional excretion at baseline and end of treatment for all-patient cohort.** Analysis of patients randomly selected from each treatment arm of AURORA 1, where data is presented as LS means of fractional excretion at baseline and at EoT. eGFR, estimated glomerular filtration rate; EoT, end of treatment; LS, least squares; SE, standard error.

|  | **Fractional Excretion (LS Means (SE))** | | | | | | | |
| --- | --- | --- | --- | --- | --- | --- | --- | --- |
| **Analyte** | **Voclosporin** | | | | **Control** | | | |
|  | **Baseline** | **N** | **EoT** | **N** | **Baseline** | **N** | **EoT** | **N** |
| **All-Patient (Responders, Non-Responders and eGFR decrease)** | | | | | | | | |
| Magnesium | 1.39 (0.148) | 53 | 2.13 (0.283) | 41 | 1.65 (0.145) | 55 | 2.03 (0.264) | 47 |
| Sodium | 0.78 (0.052) | 53 | 1.00 (0.201) | 41 | 0.86 (0.051) | 55 | 1.16 (0.188) | 47 |
| Potassium | 8.44 (0.484) | 52 | 10.50 (1.448) | 40 | 8.49 (0.479) | 53 | 9.54 (1.350) | 47 |

Supplemental Figure 1. Patient subgroup flowchart.

Treatment responders were defined as those with >50% reduction from baseline in UPCR at Week 52; all patients not achieving at 50% reducing in UPCR, including patients with missing data, were considered nonresponders. ^a^eGFR decrease defined as ≥30% reduction from baseline in eGFR during the study, confirmed based on two consecutive eGFR measures.
